# How to Make a Synthetic Multicellular Computer

**DOI:** 10.1371/journal.pone.0081248

**Published:** 2014-02-19

**Authors:** Javier Macia, Ricard Sole

**Affiliations:** 1 ICREA-Complex Systems Lab, Universitat Pompeu Fabra, Barcelona, Spain; 2 Institut de Biologia Evolutiva, UPF-CSIC, Barcelona, Spain; 3 Santa Fe Institute, Santa Fe, New Mexico, United States of America; Imperial College London, United Kingdom

## Abstract

Biological systems perform computations at multiple scales and they do so in a robust way. Engineering metaphors have often been used in order to provide a rationale for modeling cellular and molecular computing networks and as the basis for their synthetic design. However, a major constraint in this mapping between electronic and wet computational circuits is the wiring problem. Although wires are identical within electronic devices, they must be different when using synthetic biology designs. Moreover, in most cases the designed molecular systems cannot be reused for other functions. A new approximation allows us to simplify the problem by using synthetic cellular consortia where the output of the computation is distributed over multiple engineered cells. By evolving circuits in silico, we can obtain the minimal sets of Boolean units required to solve the given problem at the lowest cost using cellular consortia. Our analysis reveals that the basic set of logic units is typically non-standard. Among the most common units, the so called inverted IMPLIES (N-Implies) appears to be one of the most important elements along with the NOT and AND functions. Although NOR and NAND gates are widely used in electronics, evolved circuits based on combinations of these gates are rare, thus suggesting that the strategy of combining the same basic logic gates might be inappropriate in order to easily implement synthetic computational constructs. The implications for future synthetic designs, the general view of synthetic biology as a standard engineering domain, as well as potencial drawbacks are outlined.

## Introduction

A fundamental trait of biological systems is their capacity to perform computations [1]. Although cells are composed of molecules and their viability relies on extracting and using energy to maintain them, they are not “just” matter and energy. Information, and how it is processed and used, is an essential ingredient of biology. Adaptation to environmental signals requires the processing and proper output to incoming information. This is of no surprise when we consider that life is strongly tied to genetic information [2]. Similarly, a computational picture of biological systems is at the core of important, unanswered questions on how organisms behave [3].

**Figure 1:**
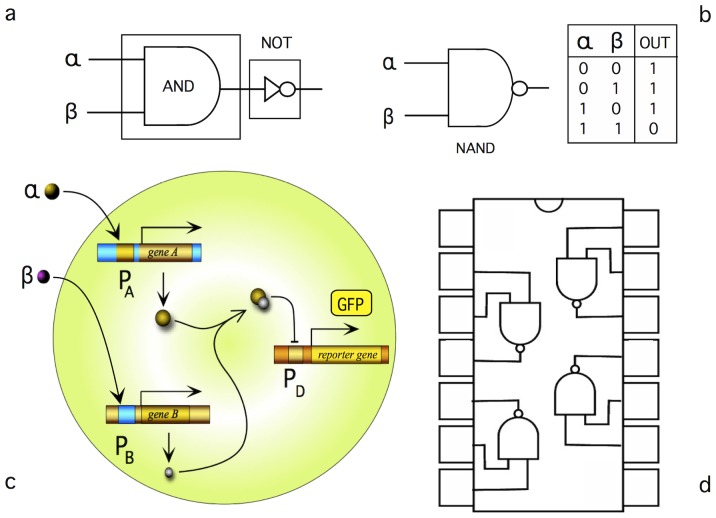
Simple logic gates can be implemented out from minimal sets of logic units. The NAND gate (a) is obtained as a sequential combination of AND and NOT gates. The compressed symbol is shown in (b) along with the truth table. An example of a synthetic implementation of the NAND logic can be made (c) using genetic regulatory elements A and B forming a regulatory heterodimer complex that prevents the expression of the reporter gene. In conventional electronics, combinations of such gates allow to construct more complex circuits and chips (d), which are then used as basic modules for further circuit designs.

How do biological systems compute? Computation is present at multiple scales, from molecules to collective decisions [4]–[10]. Developmental processes [11], [12], collective intelligence [13], [14] and complex decision-making in cells [15]–[18] can be mapped into some class of formal computational framework. Early works in theoretical biology, cybernetics, and Boolean dynamical systems widely emphasized the view of molecular phenomena within cells as the likely result of computational processes [19]–[22]. But beyond the classical theoretical approach to computation, one especially important avenue involves the engineering of cellular circuits in order to construct given computational functions or devices performing computations [23]–[31]. An example is given in figure 1, where we show the potential implementation of a simple logic gate using engineered regulatory networks. Here a NAND gate is built by combining a few basic components. Two input molecules (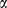 and 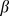) can be sensed by appropriate receptors or simply diffuse into the cell where they interact with operator sites. Only in the absence of both signals the output is produced.

In this context, it has been suggested that complex computational tasks might be obtained by engineering biological structures (molecules and cells) in such a way that they can respond to given sets of inputs and generate a pre-defined output response. Using synthetic biology techniques, a great deal of examples involving logic gates and simple combinations of them have been obtained and some specific computational problems addressed (see [24], [32], [33] and references cited). Much is expected from these developments towards new approaches to complex diseases, for example. But the promise of a reliable, scalable, reusable, robust and predictable life-based technology that could allow constructing complex living machines has been shown to be much more limited than expected [34]. After a successful first wave of important results, the promise of arbitrarily complex constructs obtained in a LEGO-like fashion is far from achieved. A flexible toolbox of reusable elements is yet to be developed and all synthetic designs so far devised are lmited to specific tasks and can not be applied to other problems. In particular, the combinatorial potential implicit in standard circuit engineering has not yet been explored.

**Figure 2:**
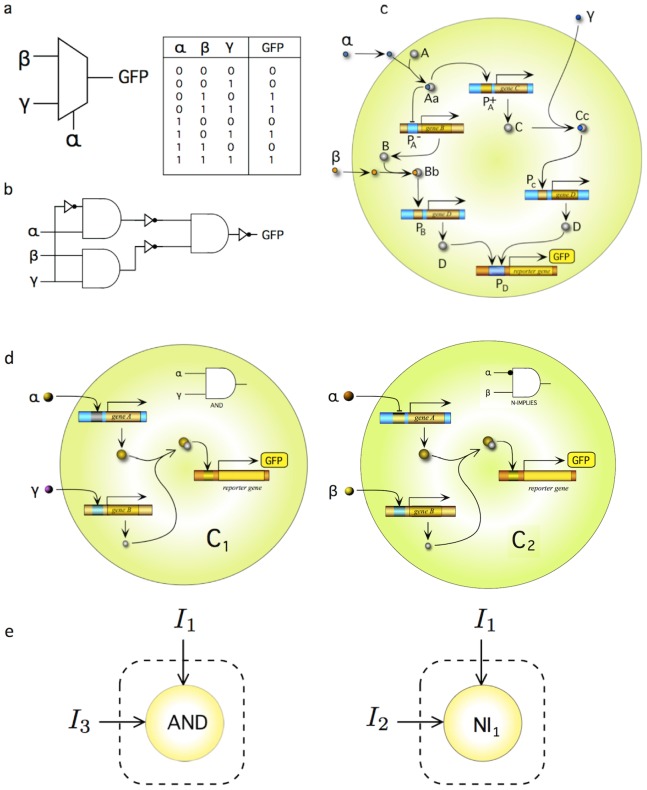
Standard and non-standard circuit design. Combinatorial circuits are constructed in conventional electronic design by using predefined gates and wiring them in order to execute a given input-output table. This is illustrated by the so called multiplexer (MUX) circuit, whose representation and logic table is shown in (a). Using AND and NOT gates, a standard implementation is displayed in (b). In (c) we show an example of a synthetic gene network implementing a single-cell multiplexer. The output signal is a GFP reporter. A very different design of the MUX system is shown in (d). Here the circuit can be easily designed by splitting the computation into two separated *and disconnected* engineered cells, both able to display the output signal. A simplified diagram that summarized the logic of (d) is shown in (e).

Although it is known from the basic theory of combinatorial circuits that some particular logic gates (such as the NOR or NAND) can be used to build any conceivable circuit, this extrapolation has failed to succeed when applied to synthetic biological designs. In that sense, although several authors have been able to build such special gates and claimed that they could *in principle* implement any potential, complex cellular circuit, the extra engineering required makes that claim far fetched. More specifically, the idea of constructing complex devices by just combining logic gates in a standard manner fails due to the so-called *wiring problem*. In contrast to electronics, where all wires are identical but physically isolated, in a cellular context each connection must be implemented by a different biochemical element, e.g. proteins. Even relatively simple devices, such is a MUX circuit (figure 2a), are difficult to obtain [35]. This circuit involves three inputs, one of which (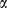) is the so called *selector signal*. As can be seen, the state of this selector element determines which one of the two inputs (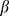 and 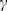) is “chosen” as the final output. Its use is widespread in electronics and it is part of many different applications. In figure 2b we show a standard implementation of this circuit obtained by connecting several NAND gates (here shown as AND+NOT elements). Despite its simplicity, this circuit requires a considerable engineering effort in order to follow standard circuit design principles within a single cell. For illustrative purposes we show a possible implementation of this circuit in figure 2c. We can easily appreciate that the internal logic of our proposed circuit requires several promoters to be connected through different molecular “wires”. Such limitations pose immediate constraints to the possibility of creating robust, scalable, and flexible devices with higher computational capacities. Hence, the development of decision-making circuits performing complex functions and, in general, the path towards living computers seems compromised by the failure of standard design principles.

One way of approaching the problem of complex wiring is to use a cellular consortium, where different types of engineered cells are at work. Such cellular consortia are common in nature [37]–[39] and provide a more flexible scenario for building complex synthetic circuits [23], [40]–[43]. Once a library of engineered cells has been constructed, it is possible to combine them in different ways to obtain different circuits. This is illustrated by the work of Tamsir et al. [44] where the authors used a set of NOR-like gates constructed on *Escherichia coli* by using two specific promoters, where the inputs and outputs are controlled by expression of different quorum-sensing molecules without cross-talk among the different cells. Here, as in conventional circuits, the colonies were spatially distributed on agar plates and connected through quorum-sensing wires. A reporter colony is used to indicate the final output response. By arranging the colonies in different spatial configurations, all of the elementary two-input logic gates can be implemented. This example provides an instance of the application of standard engineering principles to synthetic multicellular systems. However, because of its construction, it is once again limited in complexity, scalability, and flexibility.

In [45], [46] we proposed a very different approach, which we named *distributed multicellular computation* (DMC). Under this approach, circuits are also divided into different cell types, but the similarities with standard electronics ends here. Roughly speaking, we allow circuits to be broken into pieces with the component indicating output scattered over the different pieces. Several types of reporter cells can be present and do not need to be connected. Upon this assumptions, it was shown [46] that complex circuits can be built from very simple cellular consortia. Each cell requires only a small amount of engineering and additional cell-cell communication molecules can be used (but are not always needed) to exchange information among cells. Multicellular implementation is conceptually appealing. It conceals the implementation details of each encapsulated logic gate, which can be individually designed and optimized. As such, it can facilitate circuit implementation and reduce interference with the host cell’s physiology by minimizing the number of components introduced into each cell strain. Therefore, to assemble a complex multicellular circuit, the experimenter needs to be concerned with only two factors: the input-output function of each cellular gate and the output-input matching between layers. Moreover, another benefit of multicellular computing is that it allows the suppression of noise in each layer. Because the wiring-molecule output from each gate is mixed, and represents the sum over a population, spurious or ‘noisy’ responses within a small proportion of cells can be filtered out in subsequent layers [47].

Instead of using NAND gates, the reduction of wiring requirements was achieved by combining the standard AND and NOT gates with the non-standard logic gates called inverted implies (N-implies, NI). There are two possible NI logic gates (hereafter indicated as 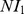 and 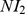) defined in tables 1 and 2 which describe a Boolean function where the underlying circuit decides which of two bits is (strictly) larger than the other. In that case the first table would correspond to 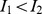 whereas the second defines 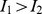. Despite these gates not being commonly used in standard designs, they have a clear biological meaning found in many regulatory genetic networks, i.e. one of the inputs triggers the expression of an output gene whereas the other blocks this expression.

**Table 1:** Truth table for a 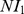 Boolean function.

*I*_1_	*I*_2_	*GFP*
0	0	0
0	1	1
1	0	0
1	1	0

**Table 2:** Truth table for a 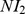 Boolean function.

*I*_1_	*I*_2_	*GFP*
0	0	0
0	1	0
1	0	1
1	1	0

Additionally, under this new approach, the gates (NOT, AND and NI) are connected in a new way. Whereas in electronics design rules try to minimize the number of gates but do not address to the number of wires or the complexity of the resulting network of connections, here the number of wires is minimized and the network of connections is reduced to a simple, fixed topology. This was experimentally implemented using engineered yeast cells, which allowed building a library of cell types that could be combined in multiple ways in order to create different types of combinatorial circuits [45], [46].

As a example, in figure 2d we present an alternative implementation for the MUX circuit based in DMC with *distributed output production*. As we can see, the two-cell implementation is made by using two *disconnected* elements, *both* being able to express the reporter gene. In electronics, it would mean breaking the circuit into two pieces; each one having a light bulb to indicate the output. Even though it makes little sense for electronic engineering, it solves a problem when dealing with a living computational device.

Considering these previous results, the goal of this paper is two-fold. On the one had we want to present a general picture of optimized circuits implementing arbitrary Boolean functions based on the combination of DMC and *distributed output production*. Such a picture would be helpful in guiding the choice and development of components and wires in synthetic constructs. On the other hand, we also want to see how far the analogies made between standard electronic circuits and their cellular counterparts can be stretched.

The previous choices make the potential set of designed circuits simple by construction. As shown below, searching for circuit designs compatible with our proposal leads to simple solutions. Such solutions largely combine a subset of logic gates that departs from the standard designs in several ways. Previous work has obtained optimal solutions in different systems [48] by using evolutionary algorithms, and the networks which evolved were simpler in some ways to hand-designed synthetic biology networks. Similarly, here we perform our analysis by means of an evolutionary algorithm exploring the space of possible designs for logic circuits. It is important to mention that our approach implicitly assumes that the underlying engineering associated with these multicellular consortia is not affected by a number of relevant problems, including cross-talk, noise, and population dynamics. Some of these problems were addressed in [45], [46] where it was shown that the DMC approach is able to overcome some of these problems. The analog, noisy, and population-dependent extensions of the work presented here will be explored elsewhere.

## Materials and Methods

### 0.1 Boolean Models of Cellular Computation

The simplest theoretical framework to define biological computation is a Boolean approximation. In such framework, the set of possible states to be observed is limited to two, i. e. 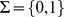. A given input string I made of zeros and ones can be written as an element of
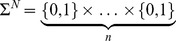
(1)


Such an input string would correspond to a set of present or absent input signals, which can be molecules but also physical variables. Let us focus on a given Boolean function 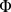 involving 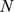 inputs and one output. This is formally indicated as a mapping
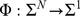
(2)


This function defines a input-output mapping between any given binary string of 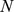 bits 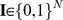 and the two possible output values 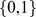. Complex circuits can be obtained out from a combination of several smaller sub-circuits called *logic gates*, and this can be done in multiple ways. These logic gates are two particularly relevant subsets of Boolean functions, namely i) the 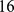 two-input one-output logic gates defining the set 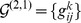 where 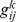 represents the *k-st* gate responding to inputs 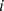 and 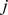, and ii) the one-input one-output gates, i. e. the set 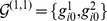 where 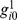 is the negation (NOT) and 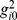 is the identity function (Id) in response to i *-st* input. It is well known that multiple subsets of logic gates can be used to implement *any* possible combinatorial circuit. These are known as *functionally complete sets* (FCSs). Typical examples of these sets are the pairs {AND,NOT} and {OR,NOT} but since the NAND and NOR gates are obtained from the combination of these previous pairs, it actually occurs that the single-function sets {NAND} and {NOR} are themselves FCSs. This statement can be proven [49] using the rules of Boolean algebra.

As mentioned above, the single-cell implementation, although possible in principle, has two drawbacks. The first involves the unavoidable design problem associated with the use of several molecular wires associated with each gene-gene connection. The second is the limited flexibility of a single-purpose design. Most typical designs cannot be recycled in any way, but a flexible and scalable system should allow for combination among components such that multiple functions could be implemented. We must keep in mind, as we have pointed out before, that the standard methods for circuit design focus on the minimization of the number of logic gates but do not pay attention to the number of wires or to the complexity of the pattern of connections. This becomes a limitation for the application of standard rules for cellular circuit design, and hence a novel methodology for cellular circuit designs seems necessary.

To reach this goal, we propose a different approach according to the following criteria.

1. the circuit can be distributed in a network of different engineered cell types (*distributed computation*),
2. the output production can take place in differently cell types simultaneously (*distributed output production*),
3. the set of *wires* connecting the different cell types must be minimal, and
4. the pattern of connections between cells must be as simple as possible.

**Figure 3:**
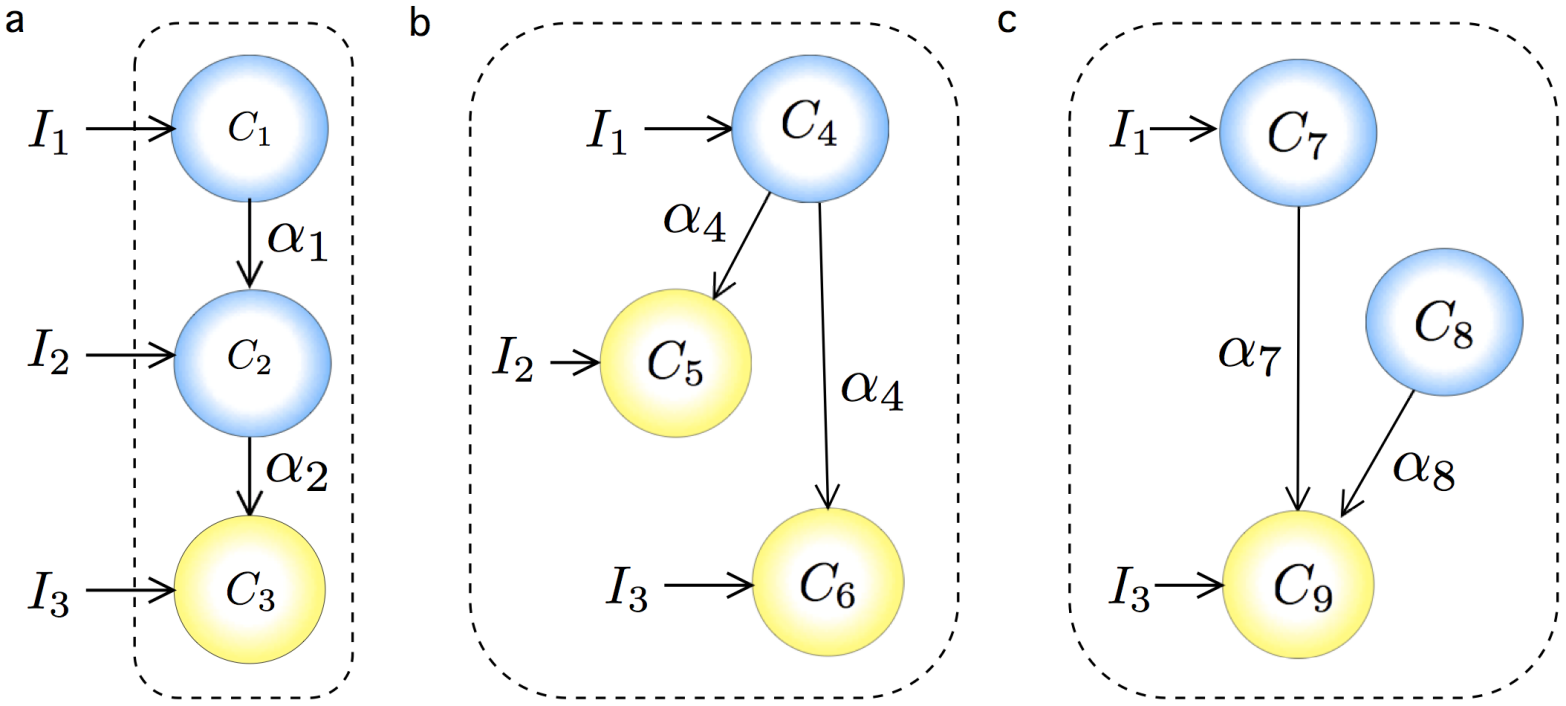
A general circuit design can be obtained by starting from a multicellular system where each virtual cell is a given logic gate. Here each engineered cell is indicated as 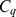 and wires generated by 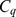 are indicated as 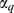. Cell 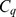 will produce an output according with the logic defined by 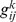. Here 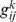 represents the *k*-th logic gate responding to two inputs, the external one 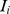 and the internal wire 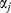 secreted by cell 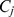. The upper layer involves single-input gates, i.e. 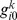 (thus only the identity or NOT are possible). Different motifs of connections can emerge according with the criteria introduced, such as independent strings of connected cells, where each cell responds to different wire (a), the same wire can be sensed by more than one cell (b), or a given cell responds to wires produced in more than one cell (c). In this last case, due to cells only can sense two inputs (one external and one internal) all wires produced in different parts of the circuit but sensed by the same cell must be implemented using the same diffusible molecule, i.e. wire 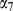 and 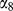 are implemented by the same molecule, which can be produced in cells 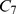 and 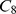 independently. This situation corresponds to an implicit implementation in 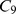 of the OR logic with respect to wires 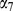 and 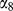. Yellow cells (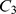, 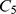, 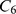 and 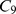) can produce independently the final output signal e.g. a GFP.

Distributed computation allows for a minimization of circuit complexity, since each cell carries a small amount of engineering, limited to implementing one given logic gate. Logic gates (cells) generate an output responding to an external input or at the most to an external input and to the output produced by other different cell, i.e. a wire, according to the logic of one of the possible functions from the set 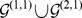. This output can be the final output of the circuit or a new wire. Of note, this assumption implies a significant difference with respect to standard methodologies because there are no hidden gates, i.e. gates responding only to internal signals (wires), typically present in standard circuits. Every feed-forward circuit implemented by our system is based on three basic motifs, shown in Figure 3. These motifs involve engineered cells (colour balls) and links 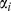 connecting them. By splitting different parts of the circuitry over different cells, we can take advantage of the intrinsic modularity of cells as units. Furthermore, distributed output production allows for a strong relaxation of wiring requirements. The connections between different cell types can be implemented by producing small diffusible molecules that can be secreted by a given cell type and sensed by another cell type. Our analysis is centred on the minimal scenario for combinational digital circuits, where feedback connections are not allowed.

### 0.2 Evolving Distributed Circuits

The efficient design of synthetic biocomputers faces a complex optimization problem. As the number of potential elements grows with circuit complexity, so does the potential number of solutions. Such combinatorial explosion can be managed by using automated methods of design [50]. This has been done in some special cases, most of which consider the analog nature of genetic systems. They include small memory devices (flip-flops, [51]), pulses and bandwidth detectors [52], and are generalized to different scenarios through simulated annealing [53], non-linear programming methods [54] standard growth and selection procedures [55], evolutionary optimization of a set of independent circuits [56], and in silico automated design inspired by standard minimization techniques borrowed from electronic design [57]. All of these methods exhibit advantages and limitations, and the predicted circuits, especially when dealing with a large number of biological parts, involve complex wiring diagrams and are not expected to be reused for multiple functions.

**Figure 4:**
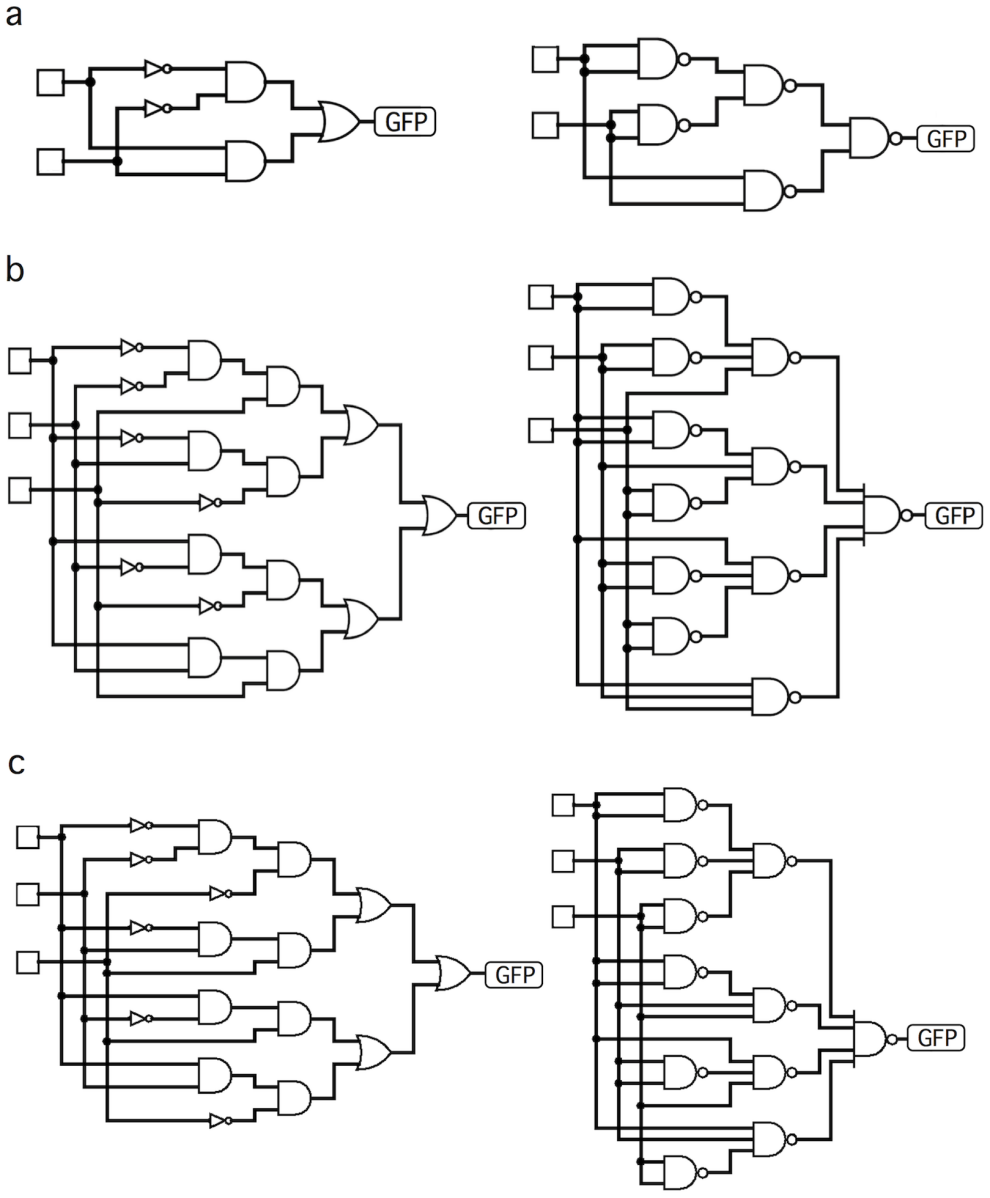
Examples of standard engineering designs of three cases studies. (a) Two-bit comparator (b) the three-bit adder and (c) a 3-bit parity circuit. Here the set of inputs appears indicated as open squares and the single-output element is marked as GFP. The left and right columns are different implementations of the same circuits design (Boolean table) but they have been constructed used diverse logic gates (left) or only using NAND gates with variable numbers of inputs. These circuits have been generated using the *Logisim* software package. Once the truth table is provided, it builds the logic circuits, either choosing the appropriate set of two-input logic gates or using just NAND gates. If the NAND gates were chosen such that they only include two inputs, the circuits would be much more complex.

In this paper we show the potential of DMC for evolving complex decision-making circuits that would be very difficult to implement using inspiration from standard electronics. Along with the previously mentioned MUX circuit, we show the result of evolving five different standard circuits of increasing complexity. These are a binary comparator, a three-bit adder, the 3-bit parity circuit (see description below), and two 4-input circuits (see below). In order to compare the expected networks resulting from electronic design principles with our (much less complex) proposed DMC constructs, in figures 4a, b, c we show their traditional implementation using logic gates. The left and right columns correspond to the implemented wiring diagrams using different one- and two-input logic gates and NAND gates with arbitrary inputs, respectively. We can clearly appreciate how rapidly the wiring complexity increases once we consider three input functions. The dramatic increase in circuit complexity is especially well illustrated by the expected designs based on NAND gates (right column). Here we allow these gates to receive multiple inputs. If we force them to include only two inputs, the number of gates and links rapidly explodes.

In order to design a given circuit implementing an arbitrary Boolean function 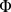, we start with a simple feed-forward architecture. We define a set of input signals 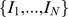 with 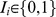 and a library 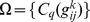 of different cells. Each cell implements the k *-st* Boolean function 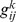, responding to both an external input 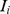 and to an internal wire 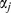, or only responding to an external input, i.e. 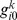.

To explore the potential sets of gates to be used in designing arbitrary circuits, we have used an evolutionary algorithm as our search engine for optimal synthetic circuits. We start with a set 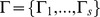 of randomly wired circuits. Here 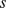 different potential solutions are considered and each circuit will have 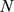 inputs and one output. The total number of possible functions implementable by this circuits is thus 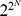. Each circuit will be composed by a random number of logic gates, which are also randomly chosen from the set 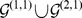 which includes 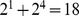 different potential choices (for general definitions, further information and classification, see [36] ). Once a given input-output function is defined, many possible combinations can implement it.

#### 0.2.1 Fitness function

Each of these circuits is characterized by a fitness function 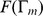. This fitness function is defined as the combination of two different terms, i.e. 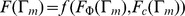. The first term, 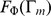, measures how good the computation of 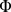 performed by the circuit 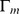 is. However, good computations are not enough but it is necessary minimize the complexity of the circuit’s design. This can be achieved in several ways. The second term of the fitness function, 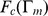, accounts for additional evolutionary scenarios satisfying the criteria presented in section 2 in order to safely translate them into an experimentally feasible construct.

Specifically, 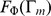 is defined as the normalized distance
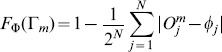
(3)where 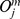 and 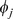 are the observed and expected outputs associated to the 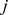-th input string (i. e. 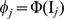), respectively. The highest value, 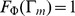, will be obtained when perfect matching is achieved.

We have explored different scenarios imposing a selection force described by the second term of the fitness function, 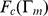, namely:

#### 0.2.2 Evolutionary algorithm

The evolutionary process starts from a set 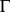 of 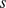 circuits. Each one of these circuits is formed by a random number 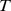 of logic gates, with 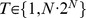, where 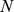 is the number of external inputs. These gates are randomly wired and the process follows several different steps.

Step 1: For each circuit the computational term of the fitness (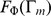) is evaluated according to expression (3) upon the different input strings 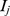.

Step 2: For all circuits with highest 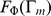 values, the second term 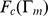 is evaluated. This criterion prevents possible biases associated with the constrained component 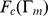, favouring good computation.

Step 3: The total fitness is calculated according to
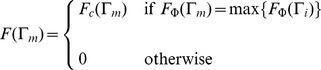
(4)where 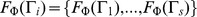. Each on of these circuits has a probability to pass to the next round proportional to the total fitness, i.e.



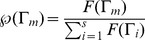
(5)Step 4: Random mutations are introduced in the circuits. Random mutations can occur at different levels, namely i) addition of a new wire with probability 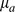, ii) deletion of a wire with probability 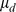, iii) addition of a new logic gate with probability 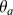, iv) deletion of an existing gate with probability 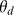, and v) modification of a logic gate with probability 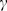. More specifically, modifications of a logic gate are implemented by inverting a randomly chosen output bit in the truth table that defines the logic function.

Step 5: In order to maintain a constant population 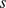 of circuits in each round, the set of circuits coming from the previous round is completed by new randomly generated circuits.

Step 6: Go to to Step 1.

In order to illustrate the potential for constructing simple, nonstandard circuits from the DMC metaphor, let us consider several representative examples. These examples belong to the most standard set of components used within electronic devices, but they are also relevant to potential applications of synthetic consortia. The circuits shown below are, among others, the minimal designs obtained simultaneously under the different scenarios described by the function 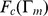 in section 3.1. Simulations were run 500 times for each 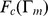 conditions with the set of parameters 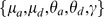 randomly chosen in the interval 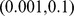 in a population of 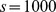 circuits. The logic circuits obtained have been tested using the 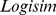 software package (see http://ozark.hendrix.edu/∼burch/logisim/ for detailed information).

1. Minimal gate number: Given a library of cells to be used, our goal is to make the total number of circuit elements as small as possible. Of note, thinking in terms of wet lab implementation, minimization of gate diversity can be useful in order to reduce the library of engineered cells. Here, 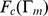 is defined as
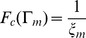
(6)where 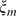 is the total number of gates forming in the circuit 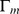.
2. Minimal wire number: The implementation of wires, i.e. connections between elements (e.g. cells), is one of the strongest limitations for synthetic designs [34], [46]. Here we minimize the total number of wires used in the circuit by defining 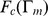 as
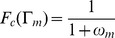
(7)where 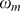 is the total number of different wires present in 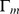.
3. Minimal complexity: This optimization procedure looks for a *structurally* minimal wiring pattern [46], [58] as the fitness function, in terms of information processed by the circuit. Roughly speaking, this complexity measure 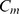 weights the contribution of modularity versus integration [58]. We define 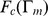 as
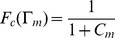
(8)where 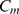 is defined as [41]

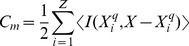
(9)Here, 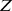 is the number of gates in the circuit and 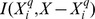 is the mutual information between the q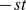 subset of the circuit formed by 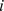 gates, i.e. 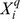, and the rest of the circuit (i. e. 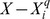). The symbols 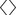 indicate the average among all possible subsets formed by 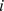 gates. Finally, the index 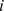 covers all possible sizes, form subsets formed by one gate (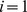) to the whole circuit (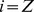).

Interestingly, as shown below, all these different constraints converge into essentially the same basic space of possible solutions.

## Results

### 0.3 Multiplexer

Our first example was the MUX circuit. The MUX circuit obtained following our method is extremely simple. The resulting construct requires just two different engineered cell types. Instead of a complex set of engineered regulatory interactions within a single cell, we can create a consortium involving just two cells. The most important feature to be noticed (aside from the simple design) is that the cells *are not connected* as a consequence of the distributed output. The whole system performs the computation and thus both pieces are required, but there is no need to couple them.

### 0.4 Comparator

In many relevant applications, a given device might need to evaluate when two or more signals are equal or not. A comparator circuit performs such an evaluation. Table 3 describes the Boolean function for a problem involving two bits (corresponding to the XNOR logic).

**Table 3:** Truth table for a Two-bits magnitude comparator.

*I*_1_	*I*_2_	*GFP*
0	0	1
0	1	0
1	0	0
1	1	1

The minimal implementation in terms of number of logic gates is a single XNOR gate. However, in a biological context, implementation of complex gates such as XOR and XNOR require the layering of multiple genetic circuits, thus necessitating substantial efforts in circuit construction and tuning [59]. As a standard alternative, this circuit can be built by combining several NOT, AND and OR gates. Comparators are a widespread component in most electronic devices and are commonly used in converters, detectors, and oscillators. Their potential for synthetic biology applications seems clear if we consider that most potential decision-making circuits performing complex tasks are likely to require this type of operation.

**Figure 5:**
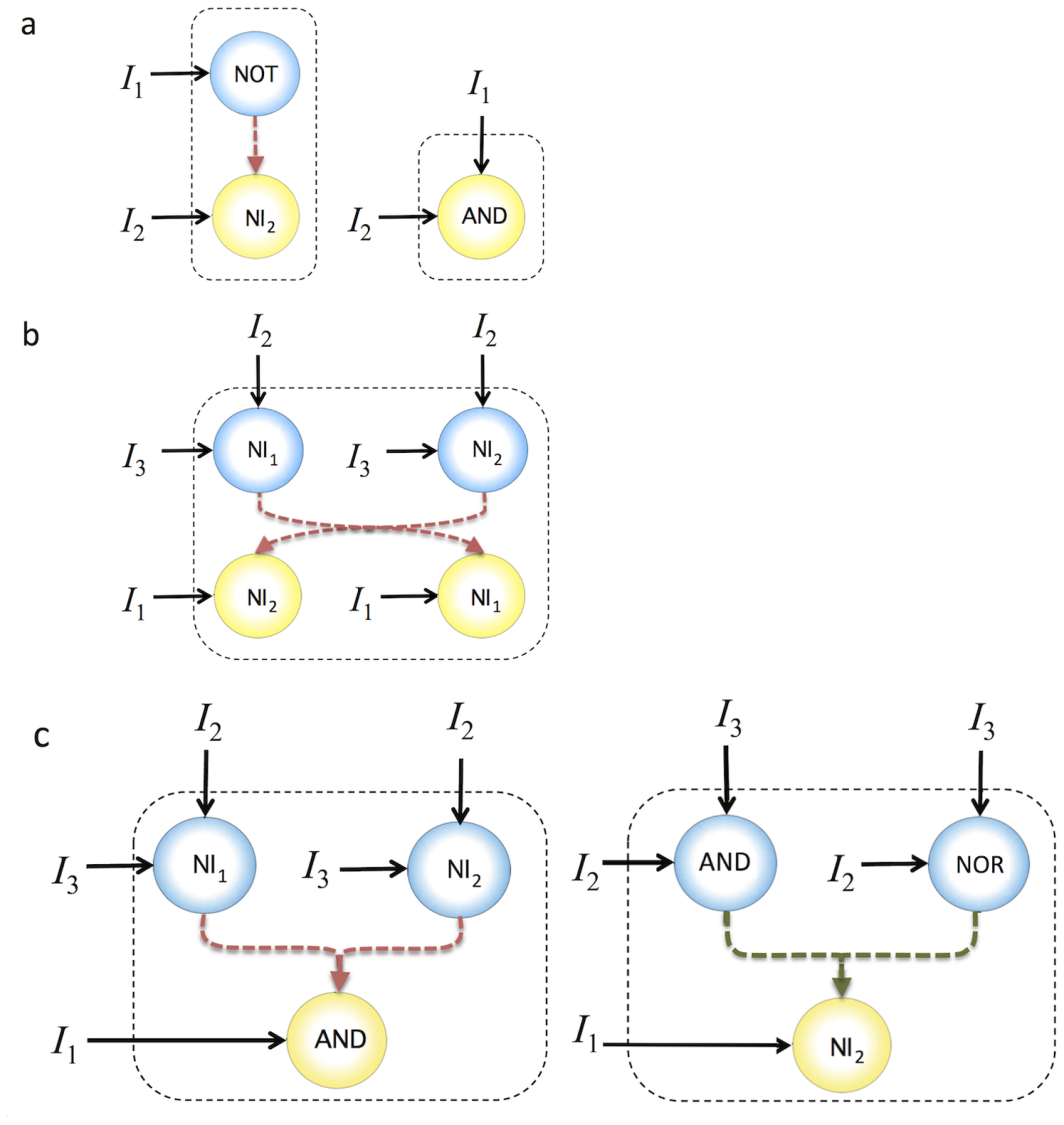
Examples DMC designs obtained by using an evolutionary algorithm to find solutions to given computational functions, as defined by Boolean tables of the examples shown in figure 4. The coloured balls represent the basic set of engineered cells indicating their internal Boolean functions. The dashed boxes indicate subsets of cells linked through the same communication signal (wire) represented by coloured dashed arrows. In (a) the binary comparator circuit is shown, after all simplifications have been performed. Figure (b) shows a DMC circuit implementing a binary three-bits adder using distributed logic. Finally, in (c) we display the minimal three-bit parity circuit is shown. Although the standard circuit is quite complex, a cell consortia involving six different cell types is enough to implement this complex function. Of note, these examples involve two different wires at the most in the most complex circuit and hence a real wet lab implementation is feasible.

Here, we have evolved this circuit imposing a minimization of circuit complexity. The minimal configuration obtained involves three different cell types implementing the AND, NOT and NI gates, and one wire (see figure 5a). As it occurs with the MUX system, two subsets of cells that do not exchange signals can be used to implement the Boolean function, whereas five NAND gates would be needed in the NAND-based logic. Of note, implementation of logic gates such as NOT, AND and NI are easier than implementation of XNOR gates [59], and the circuit implementation combining these gates (despite one wire being required) can be easier from an experimental point of view.

### 0.5 Three-bit Adder

An additional operation that is essential in most devices is the binary addition of several input numbers described by the truth table. Let us consider the three-bit adder, as defined in table 4.

**Table 4:** Truth table for a Three-bit adder.

*I*_1_	*I*_2_	*I*_3_	*I*_1_ + *I*_2_ + *I*_3_
0	0	0	0
0	0	1	1
0	1	0	1
0	1	1	0
1	0	0	1
1	0	1	0
1	1	0	0
1	1	1	1

This circuit requires a large number of gates (in both scenarios) and wires that make it almost prohibitive for real scenarios. Because of this, it provides a perfect illustration of the potential for strongly reducing circuit complexity by combining non-standard gates with distributed output production. Here the standard implementation would be highly difficult to implement, whereas the consortium solution is completely feasible (see figure 5b).

### 0.6 3-Parity Bit Circuit

Our fourth example is another three-input, one-output circuit that implements the parity bit circuit, described in table 5. An even parity bit circuit generates an output of 0 if the number of 1 *s* in the input sequence is even and 1 if the number of 1 *s* in the input sequence is odd. Because of its simplicity, parity is used in many applications where an operation can be repeated in case of difficulty, or where simply detecting an error is helpful. Parity can be used as a control to check whether the input string is the expected one or some error (wrong bit) is present. Figure 5c shows the minimal implementation found by the evolutionary algorithm. In this case two different wires are required.

**Table 5:** Truth table for a 3-Parity bit circuit.

*I*_1_	*I*_2_	*I*_3_	Paritybit
0	0	0	1
0	0	1	0
0	1	0	0
0	1	1	1
1	0	0	0
1	0	1	1
1	1	0	1
1	1	1	0

### 0.7 4-Inputs: Two Illustrative Examples

**Figure 6:**
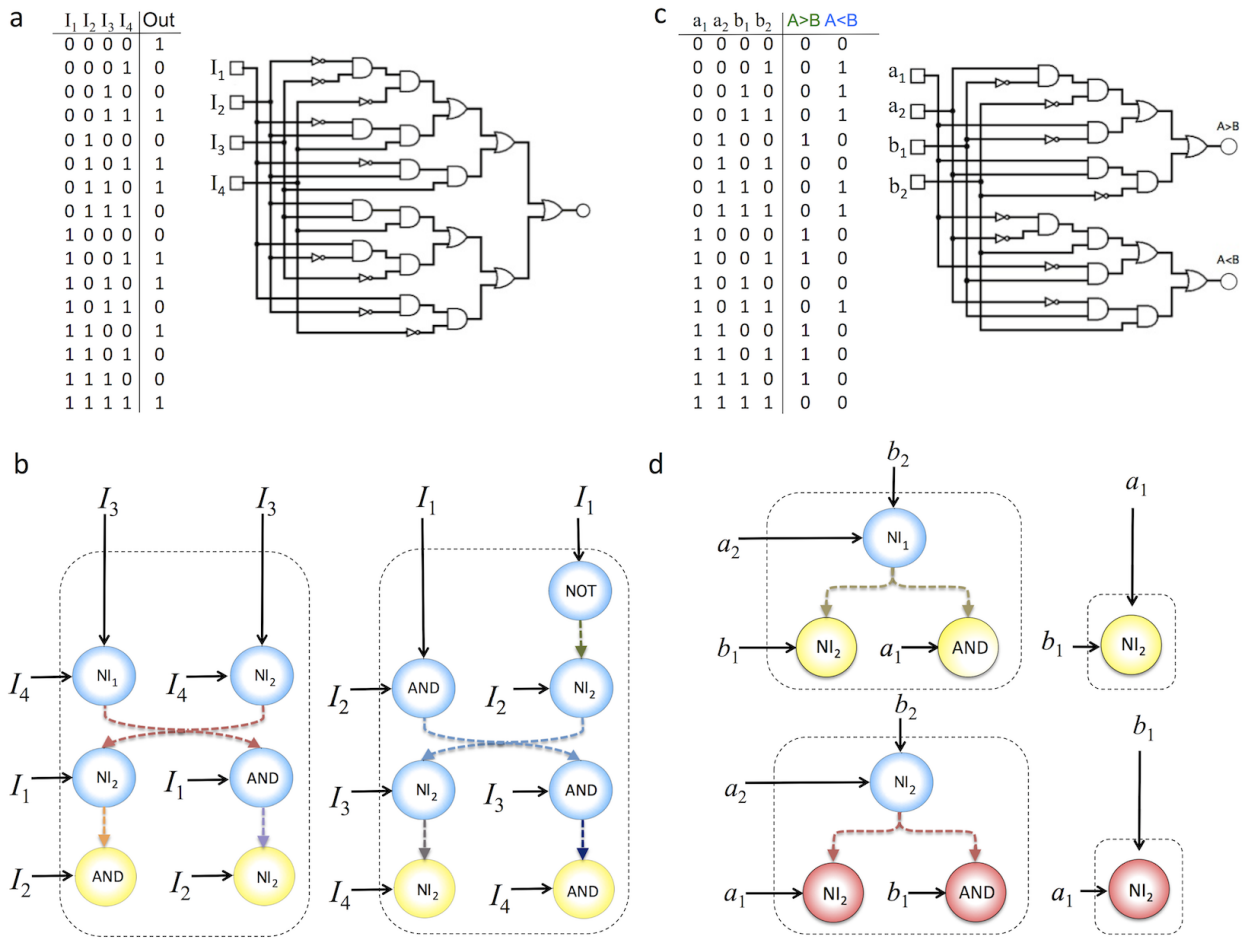
Scaling up DMC. In (a) we consider a complex, nontrivial 4-input 1-output Boolean function analysed in Marchisio and Stelling 2011 [57]. Figure shows the truth table and the standard design using two-input gates along with NOT gates. Below (b) we display a minimal circuit implementing this function. In (c) a two-bits comparator circuit is implemented according with the standard methodology, whereas in (d) an alternative design obtained by evolution is shown involving less gates an wires Only two communication signals (wires) are required for this implementation. Two colours (red and yellow) are used to indicate the two reporter molecules associated to each possible output (either 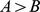 or 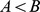).

In order to emphasize the advantages of DMC combined with distributed output production, we have analyzed one of the most complex functions involving four inputs. This function, described by the truth table shown in figure 6a, is complex due to the fact that the canonical form does not allow for Boolean simplifications, as analysed in [57]. The function involves a rather complex computation, which cannot be reduced nor decomposed in simpler components and, as a result, has a complex associated standard circuit. Here 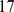 two-input gates (and four NOT gates) are needed to construct the circuit based on electronic design rules. As we can see, the number of wires rapidly explodes. Such circuit complexity makes a mapping between these designs and a synthetic construct highly unlikely to even be possible.

Again the solutions found by using our evolutionary algorithm minimizing the number of wires involve a reduced number of wires and cells. Figure 6b shows the minimal circuit obtained, involving seven wires and thirteen cells, all of them with the minimal engineering required for our method. This is the worst case scenario that can be found. Another circuit of great importance in electronic engineering, the so called two-bit comparator, which processes 4-bit input strings and has a two-bit output, is shown in figure 6c. The standard circuit is again very large, with 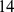 two-input gates and four inverters. In figure 6d the corresponding DMC design is shown, requiring only eight cells. Of note, it is interesting to see that the minimal circuits are formed by a non-standard combination of AND and N-Implies gates. The large number of the later type is a characteristic trait of DMC evolved circuits, as shown below.

### 0.8 Bias Towards Non-standard Gates

A more general analysis has been performed by considering the whole set of possible random 3-input, 1-output functions. Our evolutionary algorithm was used to search for evolved DMC designs minimizing circuit size and circuit complexity. The resulting optimized circuits involve different abundances of different gates and have different topological arrangements. Our results are summarized by means of a graph that captures both the frequency of gates used in the final design and how often they are found together within a given solution.

**Figure 7:**
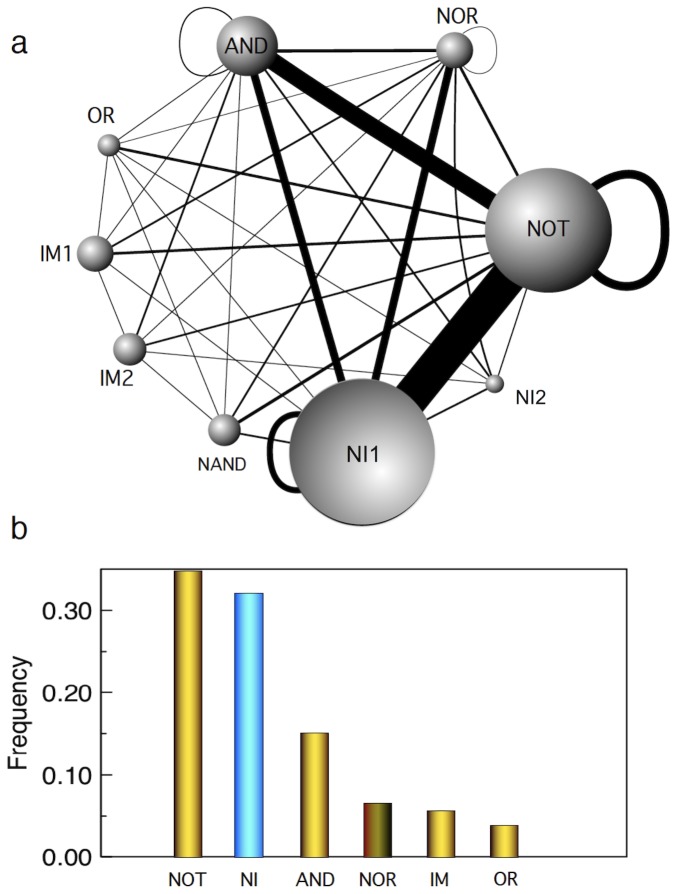
Graphs showing the frequency of gates in a MUX circuit. (a) Weighted graph showing the frequency of gates used in the generation of evolved MUX circuits under DMC. The diameter of the nodes is proportional to the frequency of the gate. Links indicate that two gates have been wired together within a circuit. The weights provide a measure of how frequently a given pair has been used. Note the disproportionate frequency of N-implies (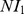) which are typically connected to NOT and AND gates, much less with NOR gates. Note also the vanishing frequency of NOR-NOR links (see text). In (b) we summarize the relative frequencies of different gates. The two N-Implies gates (here NI = N1+IN2) have been added together. These and the NOR abundances are highlighted with distinctive colours. Circuits were evolved imposing minimal number of wires.

Again, let us start with the MUX circuit, which is a good representation of a complex design. Figure 7a shows the weighted graph associated to the frequency of gates used in the generation of evolved MUX circuits imposing wires minimization. These frequencies are the average of 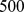 independent runs of the evolutionary algorithm evolving MUX circuits.

The diameter of the nodes is proportional to the frequency of its abundance and the thickness of the links represents the probability that two gates are present in the same circuit. The graph allows us to draw to important conclusions.

We can observe an overabundance of a few gates. The histogram shown in figure 7b allows comparing these frequencies easily. The most common 2-input 1-output gate is clearly non-standard from the point of view of engineering principles. The second is that neither combinations of the standard gates NOR or NAND seem to be very relevant here. Their frequency is much smaller than other gates, particularly the non-conventional N-IMPLIES (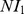), which is the most common choice, followed by AND and NOT gates. The fact that the most common gates define a non-standard set of logic functions (which has been shown to be complete, see [45]) suggests that we must consider a whole alternative landscape of combinatorial logic designs under our distributed computation paradigm.

**Figure 8:**
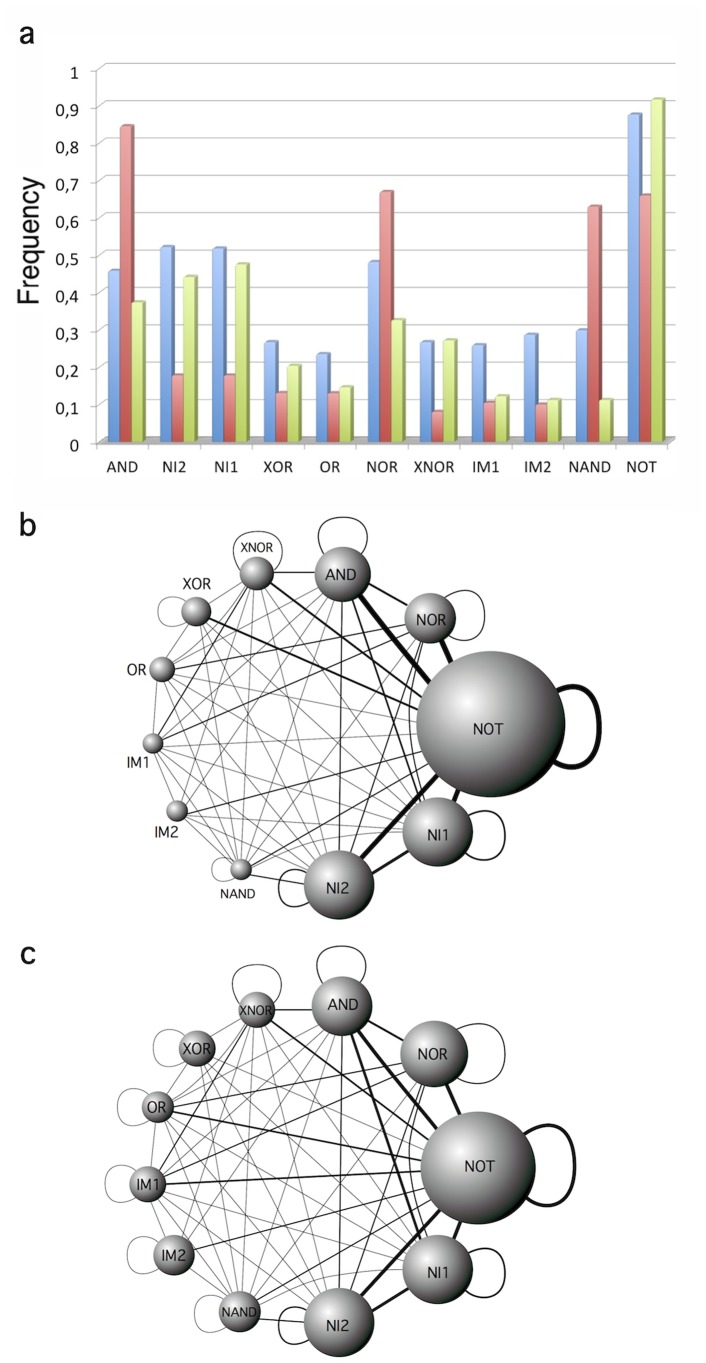
Frequency of appearance of different logic gates for circuits evolved without evolutionary pressure. In (a) the graph displays the frequency of appearance of different logic gates for circuits evolved without any specific evolutionary pressure (red), imposing minimum size (green), and under minimal circuits complexity (blue). These frequencies have been calculated as the average results for 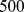 runs. In (b) the graph displays the average results for 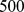 runs of an evolutionary pressure towards minimum size. The frequency of a given gate is proportional to node diameter, whereas the probability of finding two gates in the same circuit is given by the link weights. In (c) we show the corresponding results for evolved networks under minimal circuits complexity. In both cases, the most represented gates are NOT, AND, 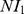, 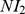 and NOR.

This analysis can be extended to the whole set of 3-input 1-output functions, i.e. for each possible function the evolutionary algorithm has found a possible solution under different constraints. This experiment has been repeated 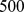 times and the frequencies shown in figure 8 are the average of these independent runs of the evolutionary algorithm. Figure 8a shows the different frequency of appearance of logic gates in circuits evolved without additional constrains (red bars), i.e. the unique imposition is a proper computation, and the corresponding results obtained by evolution imposing minimal size (green bars) and minimal complexity (blue bars). As the figure shows, the patterns are significantly different. In the absence of additional constraints, the most frequent gates are NOT, AND, and not surprisingly NOR and NAND gates. However, in the presence of additional constrains, patterns change and the abundance of NOR and NAND gates is reduced, whereas the 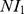 and 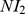 gates emerge as an alternative for improved circuits.

Despite the NOR gate is a functional complete set by itself, circuits involving only, or dominantly, NOR gates are not the optimal solutions found as the thin auto-link in the NOR node of the graphs indicate (figures 8b–c). Similar arguments can be applied to the NAND gate. Of note, the similarity between graphs obtained imposing minimal size and minimal informational complexity is remarkable, suggesting that the optimization of one aspect could be related with an implicit optimization of the other. Future work should be devoted to analyse these implications carefully.

## Discussion

The continuous advance towards the design and synthesis of biocomputers shows that there is an enormous potential for innovation [60], [61]. One of the goals of ongoing synthetic designs is the construction of complex living circuits able to perform complex decision-making tasks. Here we present a promising approach to this problem, which requires reliable and flexible approximations and the potential for extensive reuse and combination of basic units.

We have explored the landscape of circuit designs associated to a number of complex decision-making constructs based on distributed multicellular computation [45], [46]. Such circuits include a large number of potentially important scenarios, including among them many related to biomedical applications, bioremediation scenarios, and several kinds of bioengineering problems. Engineering biofilms, tissue architecture, and growing biomaterials all deal with multiple interacting cell types and it is precisely its multicellular character that makes our method scalable and easy to implement. Our results suggest that the potential associated with multicellular consortia, which have been explored in different scenarios [62]–[65], can be used for designing complex computational devices.

The results described above suggest that, according to the design rules proposed, other functional complete sets, such as AND and NI, can be more optimal for cellular implementation than the standard ones, indicating that the optimal functional complete set dependents on constraints imposed on the circuit topology. Our approximation strongly departs from standard electronic design and in doing so we are able to greatly reduce the connectivity requirements as well as provide a source of flexible combinatorial power.

Our work provides a robust approach to building more complex computations, is predictable and scalable, and opens the door to the future design of multicellular chips. It also allows for re-thinking the way cells and tissues process information beyond man-made metaphors. Its general nature makes it easily extendable to other forms of molecular interactions and model organisms or even protocellular systems [25], [66] and can take advantage of additional techniques, such as microencapsulation [67], [68]. Our results provide a proof of concept that the expectations of synthetic biology in terms of creating complex computational functions from simple ones can be reached, and allows for the exploration novel forms of optimization beyond standard engineering [69].

Despite the promising previous results obtained so far under the multicellular consortia approximation [44], [45], future work should be devoted to analyse possible drawbacks that can limit the potentiality of multicellular circuits, such as stochastic effects on low cell number populations, the impact of different growth rates in a multicellular consortia, circuit stability in long term experiments, and experimental implementation of different wire molecules. These limitations will be strongly dependent upon the specific embodiment of the future synthetic devices. For instance, the same logic circuit can be implemented by using transcriptional or post-transcriptional regulatory genetic circuits hosted in prokaryotes or eukaryotes organisms, and depending on these aspects the same circuit can behave in a different way.

Moreover, other layers of the hierarchy of computational systems, including memory and more complex integrated circuits, should also be explored. Although still far from a complex architecture that we could identify with a “computer”, our method shows enormous potential to achieve such goal.
